# Fibrous Dysplasia Presenting as an Exophytic Gingival Mass: A Rare Clinical Presentation

**DOI:** 10.3390/jcm15145659

**Published:** 2026-07-19

**Authors:** Baljinnyam Altangerel, Ok-Jun Lee, Song-Yi Yu, Ji-Yeon Kang, Eun Young Lee, Kang Hee Yu

**Affiliations:** 1 Department of Dentistry, College of Medicine, Chungbuk National University, Cheongju 28644, Republic of Korea; baljinnyam855@gmail.com (B.A.); ley926@chungbuk.ac.kr (E.Y.L.); 2 Department of Pathology, College of Medicine and Medical Research Center, Chungbuk National University, Cheongju 28644, Republic of Korea; ojlee@chungbuk.ac.kr; 3 Department of Prosthodontics, Chungbuk National University Hospital, Cheongju 28644, Republic of Korea; cbnuh77@naver.com; 4 Department of Oral and Maxillofacial Surgery, College of Medicine, Chungnam National University, Daejeon 35015, Republic of Korea; gon9404@naver.com; 5 Department of Dentistry, Chungnam National University Hospital, Daejeon 35015, Republic of Korea; 6 Department of Oral and Maxillofacial Surgery, Chungbuk National University Hospital, Cheongju 28644, Republic of Korea

**Keywords:** fibrous dysplasia of bone, gingiva, maxilla, diagnosis, differential

## Abstract

**Background/Objectives:** Fibrous dysplasia (FD) is a benign fibro-osseous disorder characterized by the replacement of normal bone with fibrous tissue and immature woven bone, most commonly involving the craniofacial skeleton. It typically presents as an intraosseous lesion in children and young adults. Fibrous dysplasia may rarely present with predominant gingival involvement and minimal radiographic evidence of intraosseous disease. We report an unusual case of craniofacial fibrous dysplasia that clinically mimicked an exophytic gingival mass in the anterior maxilla of a middle-aged patient. **Methods:** A middle-aged patient presented with a slowly enlarging gingival mass extending from the right canine to the left central incisor region. After being lost to follow-up for approximately 4.5 years, the patient returned with increased swelling, pain, spacing of the anterior teeth, and functional impairment affecting mastication and speech. Clinical, radiographic, surgical, and histopathologic findings were evaluated. Surgical management included excision of the lesion, extraction of non-restorable teeth, and bone grafting under general anesthesia. **Results:** Radiographic examination demonstrated minimal osseous involvement without a clearly defined intraosseous expansile lesion. Histopathologic analysis revealed irregular curvilinear trabeculae of woven bone within a fibrous stroma containing bland spindle cell proliferation, consistent with FD. At the six-month follow-up, the patient remained asymptomatic without complications and was undergoing prosthetic rehabilitation with plans for future implant placement. **Conclusions:** FD may rarely present as a predominantly gingival lesion with minimal radiographic evidence of bone involvement, posing a diagnostic challenge. Recognition of this atypical presentation is important to avoid misdiagnosis and to facilitate appropriate management through comprehensive clinicoradiologic and histopathologic correlation.

## 1. Introduction

Fibrous dysplasia (FD) is a benign fibro-osseous disorder in which normal bone is replaced by immature fibrous tissue and irregular woven bone, most often due to activating somatic mutations of the GNAS gene [1]. Clinically, patients may exhibit swelling, facial asymmetry, and cortical expansion [2]. Pathologic fractures and visual or auditory impairment can occur when the lesion compresses adjacent structures [3].

In general, FD presents in three forms—monostotic, polyostotic, and polyostotic with endocrinopathies, which can be associated with hyperpigmentation and endocrinological disorders and is called McCune–Albright syndrome. It is usually observed in children and young adults, with 75% of patients presenting before the age of thirty [4].

Fibrous dysplasia most commonly presents as an intrabony lesion [2]; however, in the present case, the lesion clinically manifested as a gingival mass, representing an extremely unusual presentation. This report aims to describe an unusual presentation of fibrous dysplasia manifesting predominantly as an exophytic gingival mass with minimal radiographic osseous involvement and to emphasize the importance of comprehensive clinicopathologic correlation in establishing the diagnosis.

## 2. Case

**Figure 1:**
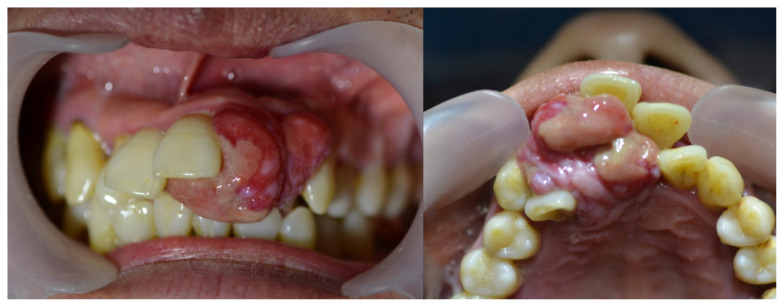
Clinical appearance at initial visit.

**Figure 2:**
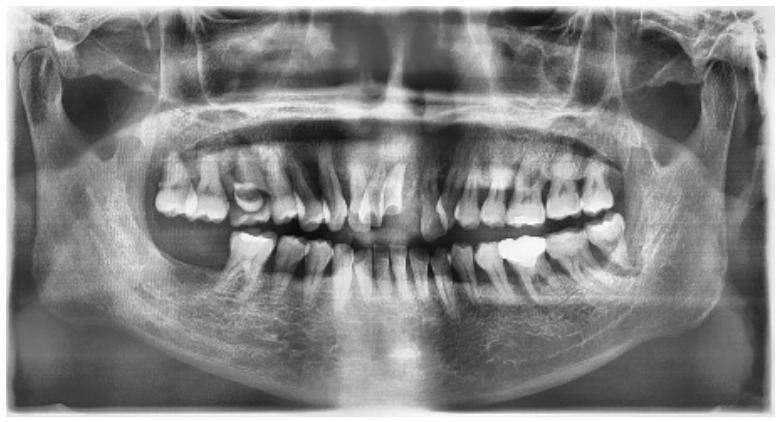
Panoramic radiography at initial visit.

**Figure 3:**
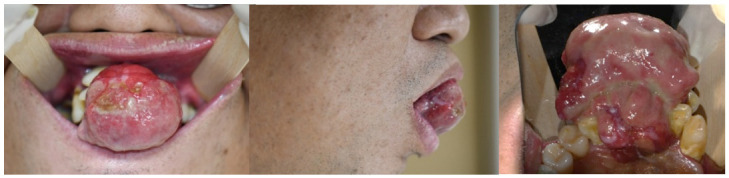
Clinical appearance 4.5 years after the initial visit.

We report a rare case of fibrous dysplasia presenting as an exophytic gingival mass. The patient exhibited a progressively enlarging mass in the anterior maxilla, extending from the right canine to the left central incisor region (Figure 1 and Figure 2). Although surgical intervention was initially planned, the patient failed to return for the scheduled appointment and re-presented approximately 4.5 years later with increased swelling and pain (Figure 3). The patient showed no clinical evidence of endocrine abnormalities or additional skeletal lesions suggestive of syndromic fibrous dysplasia.

**Figure 4:**
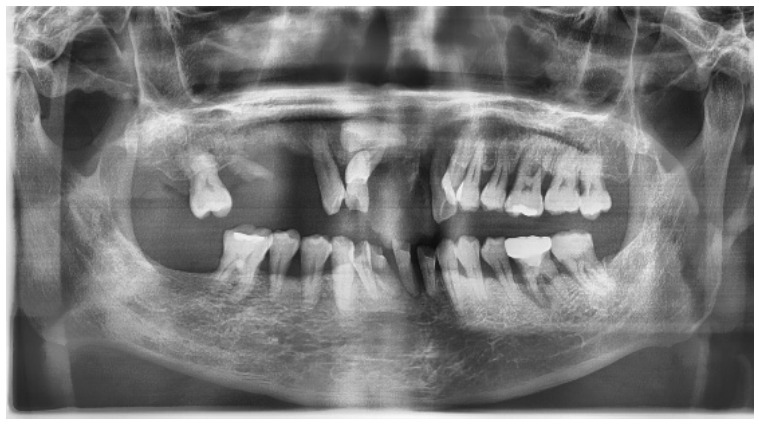
Panoramic radiography 4.5 years after the initial visit. A characteristic ground-glass appearance was not clearly identified, whereas displacement of the adjacent teeth was observed.

**Figure 5:**
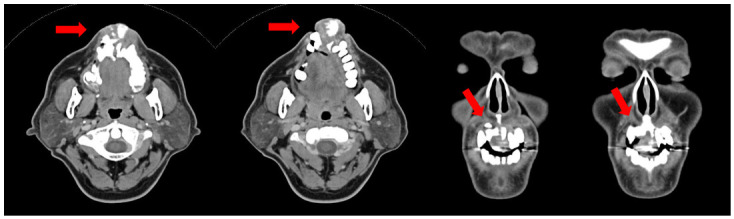
CT images suggested a periosteal or periodontal origin with minimal bone involvement.

The mass had caused spacing in both the maxillary and mandibular anterior teeth, resulting in functional impairment including difficulty in mastication and speech. Radiographic and laboratory evaluations were subsequently performed (Figure 4 and Figure 5). Computed tomography demonstrated focal cortical erosion with loss of cortical continuity at the lesion site. The lesion was directly attached to the underlying cortical bone without extension into the medullary cavity. These radiographic findings suggested cortical surface involvement without medullary disease. These findings raised the possibility of cortical surface or periosteal involvement; however, the exact site of origin could not be determined radiographically.

**Figure 6:**
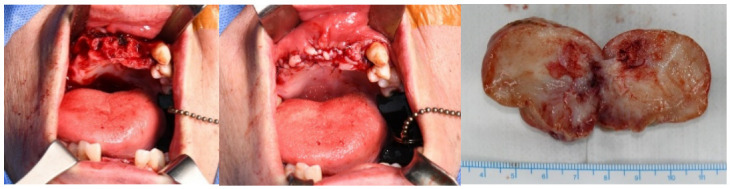
Intraoperative photograph showing excision of the mass.

The patient had a medical history of hypertension, diabetes mellitus, and hyperlipidemia, all of which were managed with medication. He was a non-smoker. Clinical examination revealed poor periodontal status, and the teeth adjacent to the lesion exhibited severe mobility and were non-vital. Laboratory investigations revealed an elevated glycated hemoglobin (HbA1c) level of 8.2%, consistent with the patient’s known diabetes mellitus. Another value including serum alkaline phosphatase, calcium, and phosphate levels, was within the normal reference ranges. Approximately five years elapsed between the initial recognition of the lesion and surgical excision. Surgical management involved excision of the lesion, extraction of non-restorable teeth, and bone grafting under general anesthesia (Figure 6).

**Figure 7:**
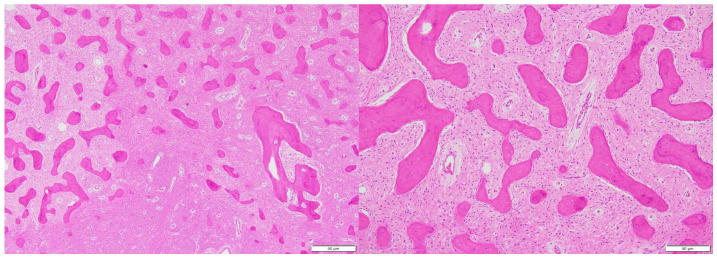
H&E staining (left: ×40, right: ×100) demonstrating irregular curvilinear woven bone trabeculae in a fibrous stroma composed of benign spindle cells, consistent with fibroblast and ropy collagen.

Histopathologic analysis demonstrated characteristic features of fibrous dysplasia, including a lesion composed of bone and benign spindle cells. Irregular, curvilinear trabeculae of woven bone were observed within a background of bland spindle cell proliferation (Figure 7). The trabeculae exhibited the characteristic “Chinese character” configuration and blended imperceptibly with the surrounding fibrous stroma without conspicuous osteoblastic rimming, findings that are considered characteristic of fibrous dysplasia. No encapsulation, peripheral maturation pattern, cementum-like calcifications, or other histopathologic features suggestive of peripheral ossifying fibroma or other benign fibro-osseous lesions were identified. Taken together, these microscopic findings strongly supported the diagnosis of fibrous dysplasia despite the atypical radiographic presentation.

**Figure 8:**
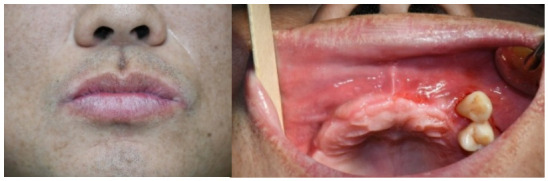
Postoperative clinical appearance.

**Figure 9:**
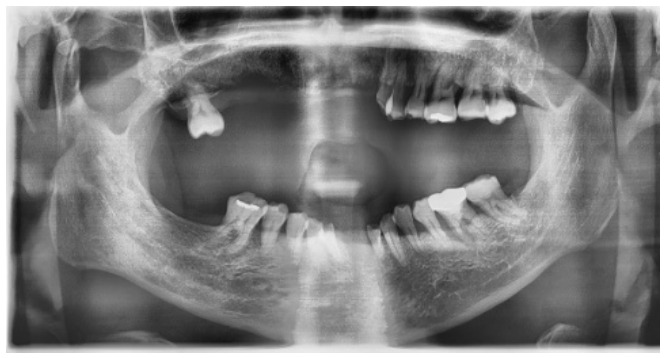
Postoperative panoramic radiography.

At the six-month follow-up, the patient remained asymptomatic with no reported complications (Figure 8 and Figure 9). The patient is currently using a removable partial denture and is planning to undergo implant placement in the future.

## 3. Discussion

Epidemiologically, FD accounts for about 2.5% of all bone tumors and >7% of benign bone tumors [5]. It is caused by postzygotic activating variants in GNAS, producing a mosaic distribution and a broad phenotype ranging from incidental lesions to severe craniofacial deformity and multisystem disease in McCune–Albright syndrome [6]. In a diagnostic utility study, an overall GNAS mutation positivity rate of around 72% has been reported in fibrous dysplasia cohorts, reflecting mosaicism and technical factors (sampling, decalcification, assay sensitivity); therefore, a negative result does not exclude FD [7].

FD involves the facial and cranial bones in nearly 50% of polyostotic FD patients and in 10–27% of monostotic FD patients [8]. Peak incidence of FD is the second decade of life, and they occur unilaterally and mostly in the maxilla [9]. In a systematic review/meta-analysis including 1260 patients, the maxilla was the most affected facial bone (41%), and facial asymmetry was the chief complaint [10]. Jaw involvement is clinically important because lesions can alter occlusion and dentition, affect the mandibular canal and cranial nerves, encroach on nasal and orbital structures, and complicate dental procedures through altered bone quality and vascularity [11]. In this case, the patient presented monostotic FD that was localized to the anterior side of the maxilla, which caused expansion of the gingiva and facial deformity.

The differential diagnosis of FD includes peripheral ossifying fibroma, non-ossifying fibroma, simple bone cyst, osteofibrous dysplasia, adamantinoma, low-grade intramedullary osteosarcoma, and Paget’s disease [2]. The current gold standard for the diagnosis of FD is a histologically proven fibro-osseous lesion with poorly defined margins which are confirmed by radiographic findings [4]. Histopathology characteristic features in FD are abnormal fibro-osseous tissue with irregular, undermineralized woven bone, and varying degrees of cellularity [5]. The stroma is low in cellularity, with curvilinear, irregular trabeculae of woven bone arranged in a discontinuous pattern [8]. The abnormal trabeculae are usually shorter, thinner, irregularly shaped, and more numerous than normal trabeculae. Osteoblastic rimming, although typically inconspicuous, was inconsistently observed in this case [12].

In the present case, peripheral ossifying fibroma was an important differential diagnosis because of the predominantly gingival presentation. However, the lesion did not demonstrate the well-circumscribed architecture, peripheral maturation pattern, or prominent cementum-like mineralization that are typically associated with peripheral ossifying fibroma. Likewise, no histopathologic features suggestive of peripheral odontogenic fibroma, such as odontogenic epithelial rests, were identified in the examined specimen. Ossifying fibroma and cemento-osseous lesions were considered less likely because the lesion exhibited poorly defined margins and irregular, curvilinear trabeculae of woven bone within a fibrous stroma, findings that are more consistent with fibrous dysplasia than with the well-demarcated growth pattern and progressive maturation usually seen in these lesions. In addition, there was no convincing histopathologic evidence suggestive of a low-grade osteosarcoma, such as significant cytologic atypia or infiltrative malignant growth. Taken together, the characteristic histopathologic features, interpreted in conjunction with the clinical and radiographic findings, were considered most consistent with fibrous dysplasia.

These abnormal trabeculae and the homogenous radiodensity account for the “ground-glass” appearance in FD [13]. However, CT appearance and natural radiographic progression vary with age and can change from homogeneous ground-glass to mixed radiodense/radiolucent lesions during adolescence, stabilizing in adulthood [14]. Mandibular chronic diffuse sclerosing osteomyelitis can mimic FD, but it showed more pain, swelling, trismus and imaging features including sclerosis, lysis and subperiosteal bone formation, whereas FD showed more bone expansion, tooth and mandibular canal displacement, with cortex often more continuous and clearly demarcated [15]. Benign fibro-osseous lesions such as FD are a heterogeneous number of bone anomalies that are described as hamartomatous formation of cellular fibrovascular connective tissue in medullary bone with varying amount of irregular, weakly calcified bone or cementum.

To the best of our knowledge, our review of the published literature did not identify any previous reports of fibrous dysplasia presenting predominantly as an exophytic gingival mass with only minimal radiographic osseous involvement. Although fibrous dysplasia has been reported in association with other lesions, such as hybrid lesions, these cases differ substantially from the present case in both their clinical and radiographic features. Abbas Karimi et al. reported a hybrid lesion composed of peripheral giant cell granuloma and fibrous dysplasia in the maxilla, accompanied by extensive radiopaque expansion involving the maxilla, nasal floor, and lateral nasal wall [9]. Saritha Kurra et al. reported a case of 18 years old female patient with fibrous dysplasia which was associated with a central giant cell granuloma [16]. 

Hybrid lesions associated with fibrous dysplasia, gingival enlargement is typically accompanied by underlying structural or histopathologic features consistent with a true hybrid lesion. In contrast, the present case did not demonstrate definitive characteristics of a hybrid lesion; nevertheless, a clinically evident gingival overgrowth was present. This unusual presentation highlights the rarity of gingival enlargement occurring in association with fibrous dysplasia in the absence of a true hybrid lesion and underscores the diagnostic challenge posed by such atypical manifestations. 

Treatment protocols for FD include observation, medication, and surgery. Medical treatment with bisphosphonates may have benefits including improvement of function, pain relief, and lower fracture risk in appropriately selected FD patients [17]. In a randomized, double-blind, placebo-controlled trial (n = 40) of high-dose oral alendronate in polyostotic FD, treatment reduced bone resorption markers and improved areal bone mineral density but did not significantly improve pain or functional parameters [18]. Also, the use of bisphosphonates is consistently associated with a potential risk of MRONJ. In a JOMS FD cohort exposed to bisphosphonates (n = 76), osteonecrosis of the jaw occurred in 4 patients (5.4%), with recognized risk modifiers including long-term high-dose intravenous exposure, dental infections, and dentoalveolar surgery [19]. Denosumab has a mechanistic rationale via RANKL inhibition and has shown reductions in lesion activity in small studies; however, discontinuation is associated with rebound bone turnover and hypercalcemia risk [20].

Surgical intervention, such as contouring or resection, is indicated in cases of functional compromise, progressive deformity, or significant esthetic concern. Surgery may be complicated by intraoperative bleeding due to lesion vascularity, postoperative contour asymmetry, nerve disturbance depending on mandibular canal course, and infection. Extensive lesions, particularly in polyostotic disease or MAS, are often unresectable and require repeated contouring or debulking for functional and esthetic management [14]. Meta-analytic data indicate that conservative surgery has higher regrowth than radical resection, with recurrence after conservative procedures reported as 84% in syndromic disease and 26% in non-syndromic disease [10].

In our case, the lesion presented as a large mass in the anterior maxilla, which not only caused significant esthetic concern but also exerted pressure on both the upper and lower teeth, ultimately interfering with basic functions such as eating and speaking. Peripheral ossifying fibroma was initially considered because the lesion clinically presented as a gingival mass. However, histopathologic examination demonstrated irregular curvilinear trabeculae of woven bone within a fibrous stroma with minimal osteoblastic rimming, which is characteristic of fibrous dysplasia. In contrast, peripheral ossifying fibroma typically shows cellular fibrous connective tissue containing well-formed bone or cementum-like calcifications and frequently demonstrates osteoblastic rimming.

Another unusual feature of the present case was the absence of a clearly identifiable intraosseous expansile lesion on radiographic examination. Fibrous dysplasia is classically described as an intraosseous fibro-osseous disorder that produces a characteristic ground-glass radiographic appearance. However, radiographic manifestations may vary depending on the stage and extent of the lesion. In some cases with limited involvement, radiologic findings may be subtle or inconspicuous, particularly when the lesion predominantly presents as a soft tissue mass.

In the present case, the lesion clinically appeared as a gingival mass and radiographic bone involvement was minimal. The biopsy specimen was limited to the excised lesion and did not include sufficient adjacent cortical bone or periosteum, the histopathologic relationship between the lesion and the surrounding osseous structures could not be reliably evaluated.

Although molecular confirmation of an activating GNAS mutation would have provided additional diagnostic support, the diagnosis of fibrous dysplasia was considered reliable because the lesion demonstrated characteristic histopathologic features together with compatible clinical findings. Nonetheless, the absence of molecular testing represents a limitation of this case, particularly in view of its atypical radiographic presentation, and should be acknowledged when interpreting the findings.

Most reported cases of fibrous dysplasia present as intraosseous lesions with characteristic radiographic findings, whereas gingival or predominantly peripheral presentations are exceedingly uncommon. In previously reported cases, gingival enlargement was generally associated with obvious underlying osseous involvement. In contrast, the present case manifested primarily as an exophytic gingival mass with only minimal cortical involvement on CT, making the clinical diagnosis particularly challenging. This unusual presentation broadened the differential diagnosis to include peripheral ossifying fibroma and other benign fibro-osseous lesions. Nevertheless, the characteristic histopathologic findings showing irregular woven bone trabeculae within fibrous stroma strongly supported the diagnosis of fibrous dysplasia, highlighting the importance of comprehensive clinicopathologic correlation in atypical presentations.

## 4. Conclusions

Fibrous dysplasia is a rare skeletal disorder that typically presents with bony deformities. In the present case, fibrous dysplasia presented as a gingival mass, highlighting an unusual clinical manifestation and emphasizing the importance of comprehensive clinicoradiologic and histopathologic evaluation for accurate diagnosis.

## Data Availability

The original contributions presented in this study are included in the article. Further inquiries can be directed to the corresponding authors.

## Abbreviations

The following abbreviations are used in this manuscript:

FD	Fibrous Dysplasia
CT	Computed Tomography
MRONJ	Medication-Related OsteoNecrosis of the Jaw
